# Molecular Diversity and Network Complexity in Growing Protocells

**DOI:** 10.3390/life9020053

**Published:** 2019-06-20

**Authors:** Atsushi Kamimura, Kunihiko Kaneko

**Affiliations:** 1 Department of Basic Science, Graduate School of Arts and Sciences, The University of Tokyo, 3-8-1, Komaba, Meguro-ku, Tokyo 153-8902, Japan; 2 Research Center for Complex Systems Biology, Universal Biology Institute, The University of Tokyo, 3-8-1, Komaba, Meguro-ku, Tokyo 153-8902, Japan

**Keywords:** protocells, diversity, mutually catalytic networks, resource limitation

## Abstract

A great variety of molecular components is encapsulated in cells. Each of these components is replicated for cell reproduction. To address the essential role of the huge diversity of cellular components, we studied a model of protocells that convert resources into catalysts with the aid of a catalytic reaction network. As the resources were limited, the diversity in the intracellular components was found to be increased to allow the use of diverse resources for cellular growth. A scaling relation was demonstrated between resource abundances and molecular diversity. In the present study, we examined how the molecular species diversify and how complex catalytic reaction networks develop through an evolutionary course. At some generations, molecular species first appear as parasites that do not contribute to the replication of other molecules. Later, the species turn into host species that contribute to the replication of other species, with further diversification of molecular species. Thus, a complex joint network evolves with this successive increase in species. The present study sheds new light on the origin of molecular diversity and complex reaction networks at the primitive stage of a cell.

## 1. Introduction

Diversity is one of the fascinating features of life. Diverse molecular species are encapsulated and coexist in cellular compartments; they are synthesized with the aid of catalysts for cell reproduction. Then, why do cells have so many components? This question arises because such a great diversity of molecular species is not a strict requirement for cell reproduction. Rather, it would not be conducive to realize high growth of a cell. In fact, a simple cell consisting of fewer components is generally expected to achieve rapid growth. This expectation is supported by several in vitro and in silico models. A replicator system with few components drives out a complex system with diverse components [1,2,3,4,5].

For self-sustaining reproduction, a minimum level of diversity is required to form an autocatalytic set in mutually catalytic chemical reaction networks [6,7,8,9,10,11,12,13]. However, diversification beyond the minimum requirement will decrease the growth rate: In catalytic reaction networks, a reaction rate is generally determined by the products of reactants’ concentrations. Given that the total concentrations of components are fixed, the frequency of a reaction event decreases with increasing component diversity. If there are *K* components at some concentrations, the concentration of each component decreases by ≈1/K, and the product of two concentrations decreases by $1/K^{2}$. Even if the reaction diversity increases by *K*, the total reaction rate decreases by 1/K. As a result, the growth rate decreases with increasing diversity. Hence, a minimum cell with essential components would achieve a higher reproduction rate than a complex cell with a huge diversity of components. Huge diversity would thus be evolutionarily selected out. However, cells presently comprise a huge diversity of molecules. This issue of diversity along with cell reproduction, also needs to be addressed in general for protocells [14,15,16,17,18].

In considering that cells with fewer components have higher growth, one implicitly assumes that the resources used for synthesizing each component are in sufficient supply and are always abundant. By consuming resources, cells with a minimum set of components increase their population. However, as the population increases, the resources actually get limited. Under such resource-limited conditions, cells with diverse components may have the potential to use different resources in the environment, which could help maintain the growth of cells. In this case, diversification of cellular components may be favorable. Still, if and how the diversification progresses, remains elusive.

Recently, we considered a protocell model in which catalytic molecules are replicated from resources, catalyzed by each other [19,20]. In this paper, we first reviewed the main findings of the model. By considering the consumption of resources, protocells with diverse molecular species emerged as they could utilize a variety of resources for their own growth. Under a selection pressure for the cells to grow faster, diversification of molecular species occurred when the resources were limited. We then elucidated how the number of molecular species increases with decreasing resource abundances. A scaling relation was then derived between molecule diversity and resource uptake, as the optimum diversity to achieve the maximum growth rate. The growth rate was maximized by a trade-off between the utility of diverse resources and concentration on fewer species to increase the reaction rate.

In the model, growth rate was maximized by optimizing the number of molecular species. However, how the cells diversify their molecular species was not explored in the previous study. In the present study, we investigate an evolutionary constraint for this diversification. The question we address here is how a molecule that emerged by mutation can be fixed and increases its population.

In a cell that grows and divides, fixation of new molecular species is highly probable if replication of the species is catalyzed by the remaining species. Then, diversification occurs by adding the species one by one to the existing catalytic network. As a result, the number of species increases in a cell, and constitutes a connected reaction network. In principle, such a complex network is not essential for high fitness (growth rate), but the evolutionary constraint selects such a connected network rather than disconnected ones.

Through the evolutional pathway of diversification, we point out the potential importance of parasitic molecular species by investigating diversification. In the evolutionary process studied here, diversity is increased by the initial appearance of parasitic molecular species followed by parasites to such parasitic molecular species. Later, they turn into host species with a further increase in the number of species. In this manner, the “core” reaction network is shifted from a simple to a complex network.

The paper is organized as follows. In Section 2, we introduce a simplified cell model consisting of a catalytic reaction network under multiple resources in the environment. In Section 3, we briefly review the results of our previous study [20]: diversification of intracellular components occurs by resource limitation, where a general scaling relation holds between the molecular diversity and uptake of resources. In Section 4, we then discuss an evolutionary constraint for cells to simultaneously satisfy the growth and diversification of components, and point out the relevance of complex, connected catalytic reaction networks. The summary and discussion are included in Section 5. Details of the simulation methods are given in Section 6.

## 2. Model

**Figure 1:**
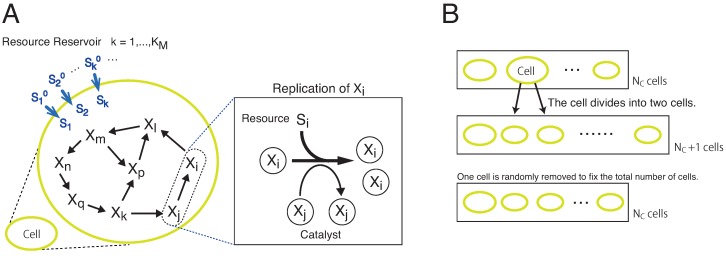
Schematic representation of our model. Our model is composed of $N_{C}$ cells. (**A**) Each of the cells contains molecules $X_{k}$ and resources $S_{k}$$\left(k=1,\cdots ,K_{M}\right)$. The molecular species form a catalytic reaction network to replicate each of $X_{k}$. For example, with the aid of the catalytic molecule $X_{j}$, the molecule $X_{i}$ is replicated when at least one resource molecule of $S_{i}$ is available in the cell. The resource $S_{i}$ is consumed to replicate $X_{i}$. Each cell takes up resources $S_{k}$ from the resource reservoir in the environment at the rate $D(s_{k}^{0}-s_{k})$. The amount of each $S_{k}^{0}$ in the environment is given by a random number $s_{k}^{0}\in \left[0,10\right]$ and is set as fixed. The parameter *D* controls the rate of uptake. By replicating $X_{k}$ with the consumption of $S_{k}$, the number of molecules $X_{k}$ increases in each cell. (**B**) A cell divides when the total number of molecules ($X_{k}$) exceeds a threshold of *N*. The contents of the cell are randomly distributed between two daughter cells. Simultaneously, one cell is randomly removed from the system to fix the total number of cells at $N_{C}$.

We adopt a cell model consisting of $K_{M}$ species of molecules and resources (Figure 1A). We denote each molecule and resource species by $X_{i}$ and $S_{i}$$\left(i=1,\cdots ,K_{M}\right)$, respectively. The molecules and resources are encapsulated in each of $N_{C}$ cells. Chemicals are well-mixed within a cell, so that sets of the amount of $X_{i}$ and $S_{i}$ for $i=1,\cdots ,K_{M}$ determine the internal state of each cell. Some of the molecular species can have a null population. Inside each cell, the molecules $X_{i}$ replicate by consuming a corresponding resource $S_{i}$, with the aid of other molecules $X_{j}$ as

(1) $$X_{i}+X_{j}+S_{i}\to 2X_{i}+X_{j}.$$

For the replication of $X_{i}$ by this reaction, one resource molecule of $S_{i}$ is needed, and the replication reaction does not occur if the amount of $S_{i}$ is less than 1. Hereafter, we denote the number of molecules and resources by small letters $x_{i}$ and $s_{i}$, respectively. The reaction coefficient is given by a catalytic activity $c_{j}$ of the molecule $X_{j}$. The reaction rate is proportional to $c_{j}$ and the product of the concentrations in concern. As $c_{j}$ is increased, the reaction rate is increased when the chemicals in concern collide. The activity $c_{j}$ is randomly determined and fixed as $c_{j}\in \left[0,1\right]$ for each $X_{j}$, throughout each set of simulations. Hence, a resource $S_{i}$ with the highest $c_{j}$ is most efficient for the replication. The reaction occurs by consuming the resource $S_{i}$. When the resources are sufficiently abundant, $s_{i}$ is given just by the environmental concentration and turns to be a parameter. Then, the replication reaction is reduced to a model with the network of hypercycles [6].

At each replication, an error occurs with a probability μ. This error corresponds to changes (replacement, insertion, or deletion) in the polymer sequence, which can alter the catalytic activity of the molecule. Here, for simplicity, for each replication of $X_{i}$, the molecule is replaced by a different molecule $X_{k}$$\left(k=1,\cdots ,K_{M};k\neq i\right)$ with equal probability $\mu /(K_{M}-1)$.

Each cell takes up resources ($S_{i}$) from its respective resource reservoir. From the external reservoir, each resource $\left(S_{i}\right)$ is supplied to each cell at the rate $D(s_{i}^{0}-s_{i})$. The coefficient *D* controls the degree of uptake rate because the resource supply is reduced by decreasing *D* (The diffusion constant across the membrane is given as *D* multiplied by the membrane width). Here, the external resources are fixed randomly as $s_{i}^{0}\in \left[0,10\right]$. We carried out stochastic simulations of the model, as detailed in Section 6. As described in Section 6, we introduced discrete simulation steps in each of which two molecules were chosen for the replication reaction (1) and the resources were updated from $s_{i}$ to $s_{i}+D\left(s_{i}^{0}-s_{i}\right)$. With this unit of *D*, D=1 indicates that the flow rate of $s_{i}$ towards $s_{i}^{0}$ is larger than the rate of the replication reactions. Here, the resources are supplied into each cell without competition among cells. We adopted this simplification as we focused on compositional diversification rather than cellular diversification.

The catalytic relation between $X_{i}$ and $X_{j}$, i.e., the catalytic reaction network, is determined by random assignment. For each pair of $X_{i}$ and $X_{j}$, the catalytic reaction path is assigned with the probability *p* (which was fixed at 0.1). Thus, each species has $pK_{M}$ reactions on average. The assignment excludes autocatalytic and direct mutually catalytic reactions. In other words, the species $X_{i}$ does not catalyze the replication of itself and that of molecules that directly catalyze the replication of $X_{i}$.

Once the catalytic reaction network is determined, it does not change throughout each simulation and is identical for all cells. Even if the underlying network is vast, each cell uses only a subset of the reaction pathways because both $X_{i}$ and $X_{j}$ must be present in the cell for the reaction (1) to occur, whereas the number of molecules in a protocell is finite as indicated below.

We assume that the volume of the cell is proportional to the number of molecules within so that the total concentrations of the molecules are conserved. The cell divides into two when the total number of molecules in the cell exceeds a given threshold *N* (Figure 1B). The molecules and resources within the cell are randomly partitioned into two daughter cells. At the division event, one cell is randomly taken out from the system and removed, to fix the total number of cells at $N_{C}$. This leads to the selection of a protocell that can grow faster under a given resource condition.

## 3. Diversification under Resource Limitation

In our previous publication [20], we investigated how diversity in cellular composition changes with the uptake rate of the resources *D* using numerical simulations of the model in Section 2. In this section, we briefly review and summarize the results.

**Figure 2:**
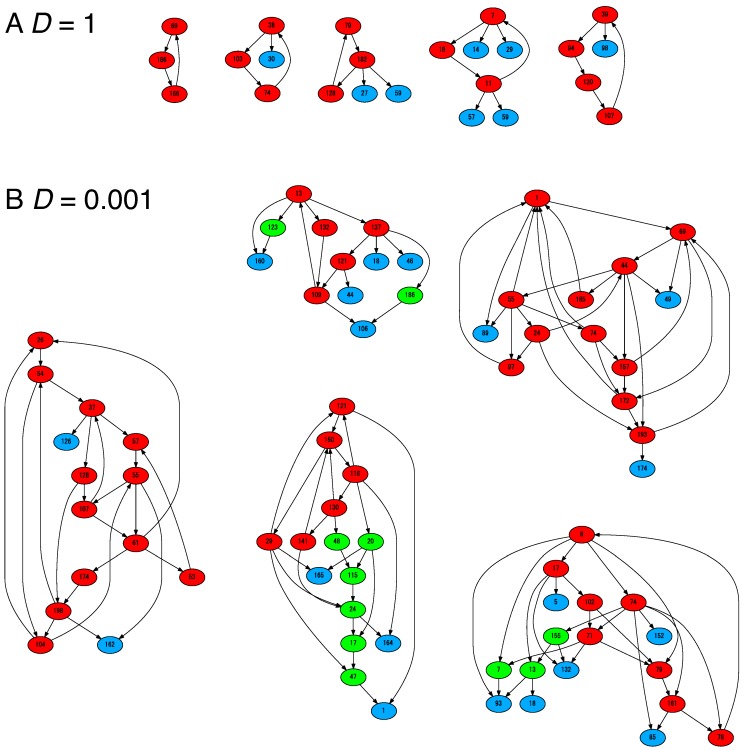
The catalytic network formed by major molecular species. The major molecular species indicates that the copy number of the species is greater than ten. For each run of simulations, almost all dividing cells have the same composition and adopt an identical network. For the parameters (**A**) D=1 and (**B**) D=0.001, such identical networks are shown to be obtained in five different underlying networks. The nodes indicate the molecular species. The arrows from *i* to *j* indicate that species $X_{i}$ catalyzes the replication of $X_{j}$. Considering the catalytic reaction network, the nodes are categorized into three types: host, sub-host, and parasite molecular species. The host species (red) belong to at least one catalytic cycle so that the set of host species is auto-catalytic. Other than the host species, the sub-host species (green) indicates that they catalyze the replication of at least one other species among the major species (but do not belong to any autocatalytic hypercycle). The parasite species (blue) indicate that their replication is catalyzed by other species, but they do not catalyze the other in turn. The parameters are $N_{C}=100$, N=1000, $K_{M}=200$, μ=0.01.

When the cells uptake resources at a sufficiently rapid rate (e.g., for D=1), three components typically dominate most of the composition for N=1000 (each representing approximately 1/3 of the molecule population). The three components, say, $X_{1}$, $X_{2}$, and $X_{3}$, configure a catalytic cycle such that $X_{1}\to X_{2}\to X_{3}\to X_{1}$, where $X_{i}\to X_{j}$ means replication of $X_{j}$ is catalyzed by $X_{i}$. This catalytic cycle warrants that each of the species has a catalyst for its own replication. Since we excluded direct mutual catalysis between *i* and *j*, this three-component hypercycle [6] is a minimum auto-catalytic set (red nodes in Figure 2A). As shown in [20], the hypercycle establishes a recursively growing state, where the composition is robust against stochasticity in the reactions and division events. Most of the other molecular species are absent, while some species can appear from time to time by replication error. Some parasitic species could increase their number on occasion (blue nodes in Figure 2A). These are catalyzed by a member of the hypercycle but do not catalyze other members. However, cells dominated by parasitic molecules cannot continue growth (In our model, any molecular species is not junk because it works as a catalyst with the counterpart molecule in Equation (1). However, the molecular species is a parasite if no counterpart molecule is present to be catalyzed. We will return to this point in Section 4.). Hence, these cells will be eliminated by selection at a cellular level. All dividing cells adopt this three-component hypercycle, and there is no compositional diversity; cells use the minimum reaction pathway to grow.

By the cell-level selection shown in Figure 1B, cells with a faster growth rate outcompete those with a slower one. By regarding a set of $\left\{X_{i}\right\}$ as a replicating entity, cells consisting of the fastest-replicating set of $\left\{X_{i}\right\}$ will take over. In our model, the growth rate of cells is defined by the replicating rates of molecules. With abundant resources, the replication rate of each molecule is determined by the product of the reactant concentrations. This product increases as the chemical abundance is concentrated on fewer molecular species. Hence, cells without other components have a higher growth rate. Thus, the three-component auto-catalytic hypercycle dominates as a result of selection at a cellular level.

However, as *D* decreases below a certain threshold $D_{c}\approx 0.01$, the number of molecular species increases, where multiple reaction pathways are utilized. As in the three-component hypercycle, the molecular species in this case also form a mutually catalytic hypercycle (red nodes in Figure 2B). The other molecular species are connected to the species in the auto-catalytic set to replicate themselves (green and blue nodes in Figure 2B). All dividing cells have approximately the same compositions. On occasion, cells dominated by parasites appear, but they cannot survive [20].

With limited resources, cells diversify their content to increase their growth rate. In this case, each replication rate is basically limited by the supply rate of its resources. Thus, cells grow fast if they utilize a greater variety of resources for their own growth. With the catalytic reaction network, cells with diverse molecular species can convert a greater variety of resources to replicate molecular species. Here, the diversity of resource species is the underlying basis for the diversification of molecular species. Therefore, cells with diverse molecular species can outcompete simpler cells.

Hence, diversification in cellular composition is a result of resource limitation. Here, whether the resources are limited or not is determined by consumption and supply rates of the resource $S_{i}$ for replicating $X_{i}$. The consumption rate is inherently proportional to the product of concentrations of $X_{i}$ and its catalyst $X_{j}$. Thus, this consumption rate decreases as the number of intracellular molecular species increases. On the other hand, the maximum supply rate is given by a constant $Ds_{j}^{0}$. The relative magnitude of the two timescales determines the degree of resource limitation.

To balance the amounts of consumption and the supply of resources, the consumption rate should decrease with the supply rate. When the consumption rate for the three-component hypercycle exceeds the supply rate, the molecular diversity starts to increase beyond three. This condition gives a transition point for *D* to diversification. With further decrease in supply below the critical point $D_{c}$, the optimal number of species for cell growth is expected to increase, as studied previously [20].

Below this optimum number of species, the consumption rate is faster than the supply rate and thus, resources are limited. Cells tend to diversify their contents to utilize a greater variety of resources for their own growth. Above the optimum number of species, the consumption rate is slower than the supply rate. Thus, the resources are abundant for the cell. In this case, cells tend to simplify their contents to increase their growth rate. Therefore, given the supply rate of resources, one expects the existence of an optimum number of molecular species to maximize the growth rate.

From this optimization for growth, one can expect a quantitative relation between the number of molecular species and the supply rate. In fact, we numerically found that the number of molecular species decreases with the parameter *D* as $D^{-\alpha }$(α≈0.5) (see [20]). This negative scaling relation is theoretically derived as follows. Let us denote the number of molecular species within a cell by $K_{M}^{*}$$\left(0<K_{M}^{*}\leq K_{M}\right)$. In the catalytic reactions, the consumption rate of resource $S_{i}$ is written as ≈$s_{i}x_{i}x_{j}$. Because the concentration of $X_{i}$ is approximately written as ≈$1/K_{M}^{*}$, we obtain

(2) $$\frac{ds_{i}}{dt}\approx -s_{i}/K_{M}^{*2}+D_{r}\left(s_{i}^{0}-s_{i}\right).$$

Thus, the steady state condition of Equation (2) is obtained as ${\bar{s}}_{i}=D_{r}s_{i}^{0}/\left(1/K_{M}^{*2}+D_{r}\right)$. As the growth rate of cells, *G*, is defined by the sum of consumption rates of resources, it is approximately written as

(3) $$G\approx \sum_{i,j}s_{i}x_{i}x_{j}\approx \sum_{i}{\bar{s}}_{i}/K_{M}^{*2}=\sum_{i}\frac{D_{r}s_{i}^{0}}{1+D_{r}K_{M}^{*2}}\approx \frac{K_{M}^{*}D_{r}s^{0}}{1+D_{r}K_{M}^{*2}},$$

where $s^{0}$ denotes the typical value of $s_{i}^{0}$. The value of the optimum number of species $K_{M}^{opt}$ to give the maximum *G* is obtained by $dG/dK_{M}^{*}=0$. Thus, from Equation (3), one gets $K_{M}^{opt}=D_{r}^{-1/2}$. Hence, the exponent −1/2 results from the second-order reaction of $X_{i}$ and $X_{j}$. The exponent changes with the order of reactions, and may also depend on the structure of the reaction network (see [20] in details).

We emphasize here that the negative scaling relation is obtained as a result of multi-level selection between molecular replication and cellular growth. At a molecular level, the coexistence of various species is possible when resources are limited. However, the argument itself does not claim that the system prefers diversification, as molecular species with a higher replication rate has higher fitness at a molecular level. Diversification of molecular species occurs as a result of selection pressure at the cell population level. The selection for growth rate causes cells to increase their components leading to an optimum level of diversity.

## 4. Evolutionary Constraints of the Catalytic Reaction Network

### 4.1. The Number of Species Is Essential for High Growth Rate

Cells with diverse species can increase their growth rates by utilizing a greater variety of resources. This mechanism explains the advantage of increasing the number of molecular species. However, how the catalytic network expands its diversity over generations is not fully explored in our previous publication [20].

Before investigating the process of diversification, we first noted that the number of molecular species is essential to convert a greater variety of resources for cell growth. The network structure itself is not necessarily essential to the growth rate as long as every species has a catalyst for its replication.

When resources are abundant, the diversity of intracellular components is decreased to realize a higher growth rate. Thus, the catalytic cycle is typically composed of a minimum three to four species. If there are multiple disjoint cycles, the cells select only a single cycle with the highest replication rate and exclude the others. Accordingly, all the nodes are connected as a single network.

**Figure 3:**
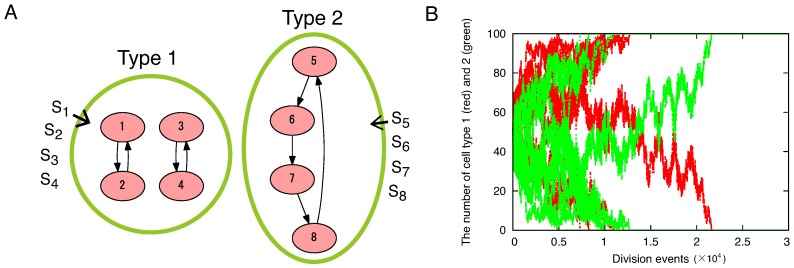
(**A**) Schematic representation of the simple model. We consider two types of reaction networks, each of which contains four molecular species. In type 1, four species $X_{1}$ to $X_{4}$ undergo the following reactions: $X_{1}+X_{2}+S_{1}\to 2X_{1}+X_{2}$, $X_{2}+X_{1}+S_{2}\to 2X_{2}+X_{1}$, $X_{3}+X_{4}+S_{3}\to 2X_{3}+X_{4}$, $X_{4}+X_{3}+S_{4}\to 2X_{4}+X_{3}$. In type 2, four species $X_{5}$ to $X_{8}$ undergo $X_{5}+X_{8}+S_{5}\to 2X_{5}+X_{8}$, $X_{6}+X_{5}+S_{6}\to 2X_{6}+X_{5}$, $X_{7}+X_{6}+S_{7}\to 2X_{7}+X_{6}$, $X_{8}+X_{7}+S_{8}\to 2X_{8}+X_{7}$. (**B**) Competition of the two types of cells. Either of the two types dominate the entire population. There is no selective advantage for either type 1 or 2. One of the two types remains by chance for each run. Red and green curves indicate the number of cells of type 1 and 2, respectively, for ten different simulation runs. In ten simulation runs, type 1 dominates the population in six runs and type 2 dominates in four runs. Parameters are $N_{C}=100$, N=1000, D=0.001, $c_{i}=1$, $s_{i}^{0}=10$ for i=1,⋯,8.

When resources are limited, a single connected network itself is not essential for high growth rate. To demonstrate this, we consider a simple model in Figure 3A. We consider two types of cells. Both types are composed of four molecular species. In type 1, the molecular species form two sets of mutually catalytic cycles. In type 2, the molecular species form a single cycle. The other parameters are identical for the two types. Thus, type 1 has two independent cycles and type 2 has a single network. For this simple illustration, the direct mutual catalytic relation (i→j, j→i) is allowed here, instead of the three-component loop in the previous section. The same argument as presented here is applied for comparison between the two disconnected three-component hypercycles and one six-component loop.

The growth rates of the two types are equal when resources are limited. Because the number of molecular species is essential for the growth rate, the structure of the catalytic network is not relevant. Thus, there is no selective advantage for a single joint network. In fact, survival of cell types is by chance, in direct competition between the two types (Figure 3B). Even the stochastic reactions result in the dominance of the population by either type, no difference is observed in selection preference between the two types. The result of this simple model suggests that the joint network is not an absolute requirement for higher growth rate.

### 4.2. Cells Diversify Their Molecular Species by Adding Species One by One to the Existing Network

As shown in the previous Section 4.1, a single joint network is not an absolute requirement for the growth rate. For example, in a huge variety of catalytic networks, one may naively expect that a set of disjoint networks can have the same growth rate as the joint network in Figure 2B. However, it is generally observed as a result of diversification in which all the nodes (species) are connected (Figure 2B).

We argue here that the joint network is obtained as an outcome of the evolutionary pathway to diversify the molecular species. In our simulation, a novel molecular species appears by errors in replication (“mutation”). The appearance of a new species by error is not sufficient for it to be fixed. The species has to increase its copy number. Otherwise, the species is diluted out by the growth of the cell.

To successfully increase its copy number, the new species should have its catalyst in the cell. Fixation of the new species is then possible if the remaining species can catalyze this new species. Thus, the catalytic network diversifies its molecular species so that it connects the new species to the existing catalytic network.

To attest this, we perform a simulation when the resources are limited (D=0.001). In the initial condition of the simulation, each of the $N_{C}$ cells has only three molecular species $X_{1}$, $X_{2}$ and $X_{3}$. The three species form the minimum hypercycle: $X_{1}+X_{3}\to 2X_{1}+X_{3}$, $X_{2}+X_{1}\to 2X_{2}+X_{1}$, and $X_{3}+X_{2}\to 2X_{3}+X_{2}$.

**Figure 4:**
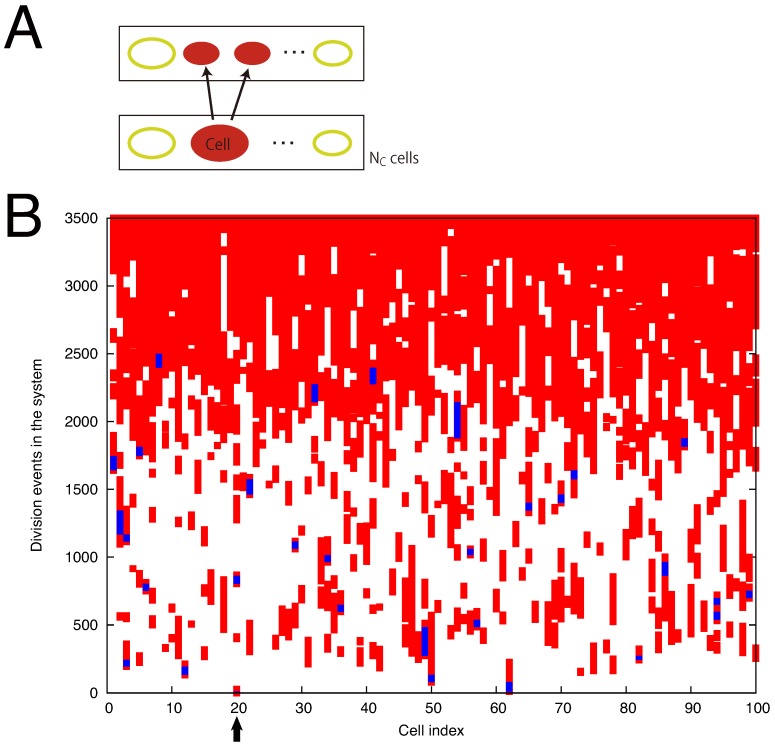
(**A**) The content of the cell is taken over by the two daughter cells. (**B**) By coloring the cells in red, one can see that all the $N_{C}$ cells are originated from one cell in the initial condition. In this sample, the initial cell at the position 20 (indicated by the arrow) is the ancestor cell. We investigate how the number of molecular species increases by tracing the content of the progeny cells (up to the 2500 division events; colored in blue) from the ancestor cell. Parameters are $N_{C}=100$, N=1000, and D=0.001.

From the minimum hypercycle, we trace the content of cells as shown in Figure 4 to examine how the cells diversify their molecular species. At cell division, the contents of a cell are taken over by the two daughter cells (Figure 4A). By coloring the daughter cells in red, we identify a single ancestor cell in the initial condition from which all the $N_{C}$ cells at the final stage are originated. In Figure 4B, all the cells are marked in red by the division events 3500. Here we also trace the content of cells along a branch of such progeny cells from the ancestral cell (up to the 2500 division events; colored blue in Figure 4B).

**Figure 5:**
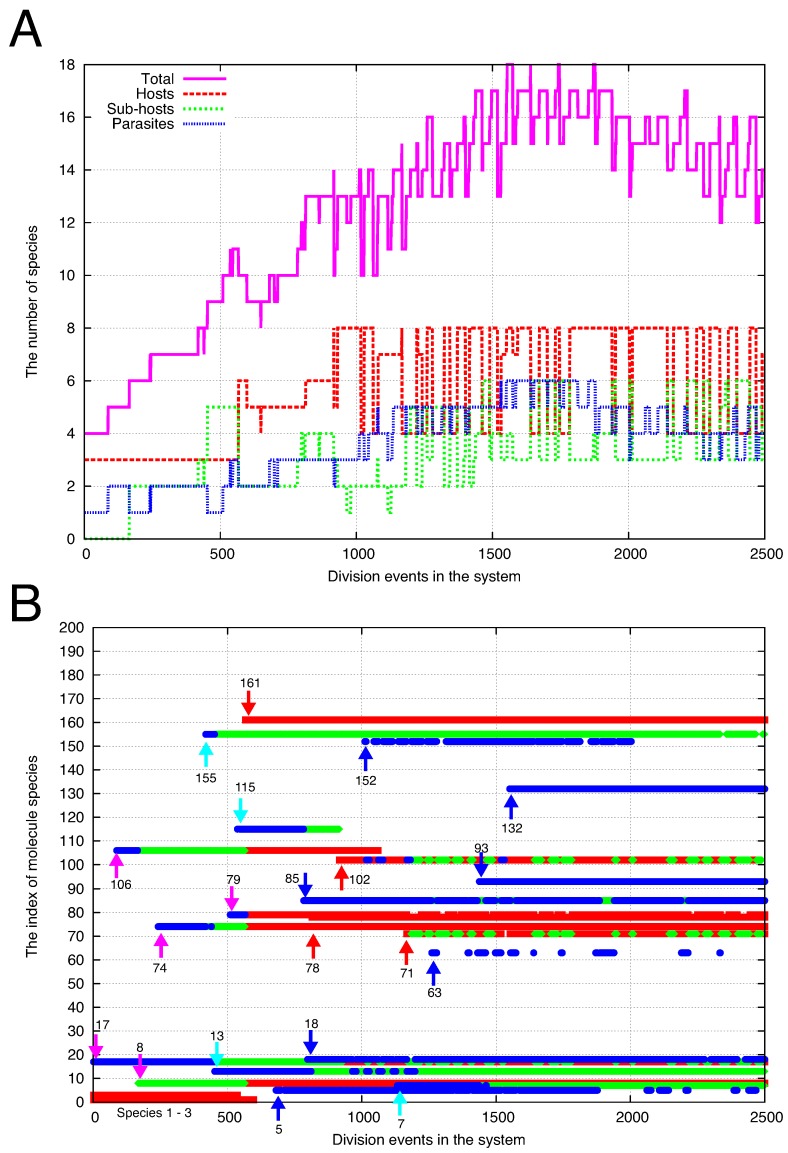
(**A**) The number of molecular species in the blue cells in Figure 4B. Other than the initial three species (1, 2, 3; here we denote the species by its number *i*, instead of $X_{i}$), the number of the major molecular species (its copy number of the species is greater than ten) in each cell is plotted against the division events in the system. The total species (magenta) indicates the total of host (red), sub-host (green), and parasite (blue) species. See Figure 2 for the definition of the species. (**B**) The indices of major molecular species in (**A**) are displayed for each cell at the corresponding time. The color of the points, red, green, or blue, indicates a host, sub-host, or parasite, respectively. Initially, the three species 1, 2, and 3 are present in the cell. Other than the three species, twenty species appear and are fixed as the major species, whereas some of them are lost. The arrows indicate the time when such species appear. The red (blue) arrow indicates that the species appears and remains as a host (parasite) in the time period, whereas the magenta arrow indicates that the species that initially appeared as a parasite (or a sub-host) changed its role to a host species (due to the appearance of other species). The light-blue arrow indicates that the species that appeared as a parasite changes its role to a sub-host species.

Figure 5A shows the number of major species in the cells along the branch. Other than the initial three molecular species ($X_{1}$, $X_{2}$, $X_{3}$), the major species is defined such that its copy number is greater than ten. The total species (magenta) indicates the number of such species. It increases from the beginning and eventually reaches a steady-state value (≈15 of this value of *D*). By looking at the catalytic network formed by the major species, we also show the number of host (red), sub-host (green), and parasite (blue) species. The host species indicate the member of an auto-catalytic hypercycle. Other than the host species, the sub-host species are defined such that they catalyze the replication of at least one other species in the major species, but do not belong to any auto-catalytic hypercycle. The parasite species indicate that their replication is catalyzed by other species, but they do not catalyze any other in turn.

Furthermore, the major molecular species in a cell are displayed in Figure 5B. Initially, the three species 1, 2, and 3 are present (hereafter, we denote the species by its number *i*, instead of $X_{i}$) and form a minimum hypercycle. Thus, the three species work as hosts and are marked by red points. Shortly thereafter, the species 17 appears as a parasite (with a blue point). Then, the species 106 also appears as a parasite. The third species 8 is fixed as a sub-host (with a green point) as it catalyzes the replication of 17. Simultaneously, the species 106, originally a parasite species, changes its role to a sub-host because it catalyzes the replication of 8 (the color of the points at species 106 changes from blue to green by the appearance of the species 8 in Figure 5B).

As the diversity increases by fixing the parasite and sub-host species, a change in host species occurs. By the emergence of species 161, several species change their role to host species (at around 500 division events). Even though the initial three species are almost simultaneously lost from the cells, the number of host species increases by successive transformation to host species from parasites. Then, most of the new species afterward are fixed and keep their role (shown with red and blue arrows), whereas some of them can be lost.

At the initial stage, all the new species start as a parasite or a sub-host species (as shown with magenta and light-blue arrows). In fact, most of the new species initially emerged as parasites. To start as a host species, the new species has to catalyze the replication of existing host species. However, the diversity of the host species is initially low due to which the probability of the new species catalyzing the host species is quite low. Hence, the new species has to start as a parasite species to the existing host species, or as a parasite to a sub-host species, i.e., a parasite to the original parasite species.

One can roughly estimate the probability with which a new species can be initially introduced as a host species. The catalytic reaction path is assigned with probability *p* (which was fixed at 0.1) for each pair of $X_{i}$ and $X_{j}$. Thus, the new species can catalyze one of the remaining host species with a probability p/2 on average (the factor 1/2 is added because only one of the two reactants works as a catalyst). By denoting the number of remaining host species as $K_{H}$, the probability that the new species can catalyze at least one host species is estimated as $pK_{H}/2$.

After fixation of species 161 in Figure 5B, the number of host species ($K_{H}$) was approximately 5 or 6 between the division events 600 and 900. Thus, the probability $pK_{H}/2$ is estimated as 0.25–0.3. In fact, one species (78) is introduced as a host whereas three species (5, 85, 18) are introduced as parasites. Thereafter, fixation of 102 further increases the number of host species to eight, which increases the probability of the appearance of host species later.

**Figure 6:**
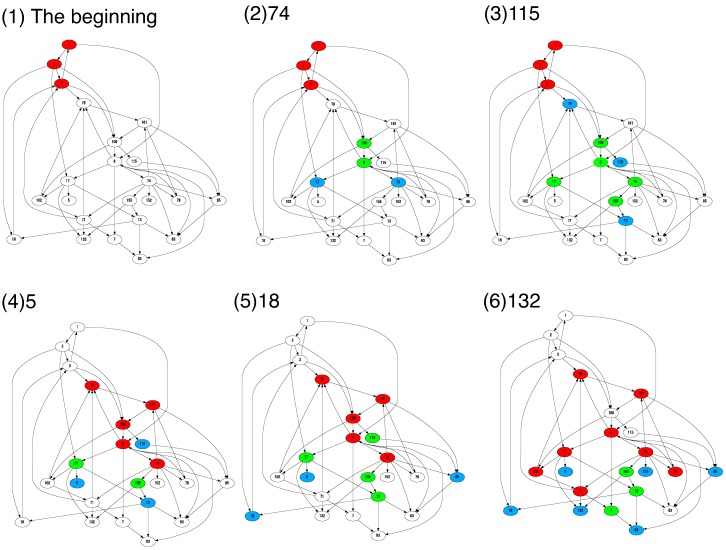
The effective catalytic network is shown at the fixation of several molecular species in Figure 5B. The red, green, and blue nodes correspond to the host, sub-host, and parasite species, respectively. The absent species at each time are represented by white nodes. For (2) to (6), the number indicates the index of the newly fixed molecular species.

To further visualize the process of diversification, we show the effective catalytic network by coloring the existing molecular species in Figure 6. The underlying catalytic network is formed by 23 potential molecular species, which appeared at some generation in Figure 5B. Here, the absent species at each generation are represented by white nodes.

As explained above, the new species initially appear and work as a parasite or sub-host species (Figure 6 (1) to (3)). As diversity increases, several species turn to be host species and a change of the “core” network occurs [(4)]. With a successive increase in host species, the molecular species further diversify and a complex joint network evolves [(5) to (6)].

From this example, one can see that the cells fix their new species one by one to simultaneously meet the requirements of growth and diversification. This evolutionary constraint suggests a potential of “parasitic” molecules. Typically, such species are considered cheaters because they are not beneficial for maintaining the “core” network. On the other hand, the species could be considered a stepping-stone toward diversification when the resources are limited. Here, we explained how diversification progresses using an example. This process of evolution through parasites is general as far as we checked five examples in the present simulation. We also show another example in a Supplementary figure.

Here, the emergence of a new species is less plausible in a disjoint network. Such fixation requires construction of another catalytic cycle from scratch. To keep the number of the new molecules against decreases by cell dilution, another mutation that catalyzes it is necessary, which hardly occurs. Then, the mechanism of connecting the species is more plausible than constructing an auto-catalytic cycle from scratch. In other words, a single connected network is more evolutionarily achievable than disjoint networks, even if their fitness (growth rate) is identical.

## 5. Discussion

Cells, in general, contain a huge variety of chemicals. In contrast, for a given environment, cells with few components can grow faster. To resolve this apparent contradiction, we investigate how the cellular composition diversifies in a cell model consisting of a catalytic reaction network. As resources are limited, the number of coexisting molecular species increases with which a variety of resources is converted to maintain their growth. By studying an example of diversification in detail, we show that the diversity is increased by the initial appearance of parasitic species followed by parasites to these parasitic species. Later they turn to be host species with further acquisition of novel molecular species.

Our model assumes that each molecular species $X_{i}$ is replicated by consuming each resource species $S_{i}$. This diversity of resource species in the environment is the underlying basis for the diversity of cellular components. In this sense, the model is similar to the GARD model [10], a kinetic model for homeostatic-growth and fission of an assembly of compositional lipids. It assumes biased accretion kinetics of molecular assemblies in diverse environmental molecules. In the growth of this assembly, the information of its composition (different types and quantities of molecules within an assembly) is transferred throughout generations. It is thus important to study the present diversity transition and scaling relation in the GARD model as well. The present result suggests an increase in the compositional information under resource limitation. Recall that the information encoded in the composition is different from that encoded in RNA as the combinatorial diversity of sequences. Still, it is interesting to note that under a limited flow of monomer resources, the sequence of catalytic polymers increases their complexity as has been shown recently [21].

In the present cells, all the diverse resources are not directly provided from the environment. Instead, most substrates for each metabolic reaction are given by components that are products of intracellular reactions. Typical bacteria only need a source of basic elements (Carbon, Nitrogen, Phosphorus, Sulfur,...) for their growth. Although the resource species are few, they are often decomposed by catabolic reactions, leading to diverse internal components. Then, through multi-body reactions, complex metabolic reactions follow with anabolic reactions. It will be interesting to extend the present study to include such multi-body reactions of polymers, and to understand the relevance of complex metabolic reaction networks for survival under resource-limited conditions.

In addition to the limitation of resources, competition between cells also provides a driving force for the diversification of cell types, which has been reported elsewhere [19]. Also, in this case, cells diversify their molecular species in order to use a variety of limited resources for their own growth. However, cells with different components can use less-competitive resources so that they can increase their population. As a result, different types of cells appear in which different sets of molecules form different catalytic networks, and they coexist in the cell population.

The diversification of molecular species in our study is reminiscent of niche differentiation in the field of ecology [22,23]. When species differentiate to specialize for each niche, their competition is relaxed, so that their coexistence is easier. However, there exists one important difference. A cell is a unit for selection, whereas an ecosystem itself is not a unit for selection as it does not reproduce. The ecosystem does not have an explicit selection pressure to grow faster. In contrast, in the present study, the diversification of molecules is a result of multi-level selection favoring a higher growth rate of a cell and higher replication of molecules. This multi-level evolutionary pressure leads to the formation of complex joint networks. This is a unique feature of cell systems with multi-level evolution [24,25].

## 6. Materials and Methods

We carried out simulations as follows. We introduced discrete simulation steps and for each simulation step, we repeated the following procedures. For each cell *q*$\left(q=1,\cdots ,N_{C}\right)$, we chose two molecules from the cell. If the pair of molecules, $X_{i}$ and $X_{j}$, were a replicator ($X_{i}$) and a catalyst ($X_{j}$), the replication of $X_{i}$ occurred with the given probability ($c_{j})$ when $s_{i}^{q}\geq 1$. $s_{i}^{q}$ is a continuous variable denoting the amount of the resource required to replicate $X_{i}$ in the cell *q*. When replication occurred, we added a new molecule of $X_{i}$ into the cell. Simultaneously, we subtracted one resource molecule of $S_{i}$ to make $s_{i}^{q}\to s_{i}^{q}-1$. With a probability μ, we added a new molecule of $X_{l}$$\left(l\neq i;s_{l}^{q}\geq 1\right)$, instead of $X_{i}$. If the total number of molecules in a cell exceeded the threshold *N*, the cell divided into two cells. We then distributed the contents randomly into the two cells. At the same time, we removed one cell to fix $N_{C}$. We then updated each $s_{i}^{q}$ to $s_{i}^{q}+D\left(s_{i}^{0}-s_{i}^{q}\right)$$\left(i=1,\cdots ,K_{M}\right)$. 

## Supplementary Materials

Supplementary File 1
