# Supplementary material for: Molecular Diversity and Network Complexity in Growing Protocells
Source: Life (Basel). 2019 Jun 20;9(2):53. doi: 10.3390/life9020053 (PMC6617351; doi:10.3390/life9020053)
Supplement: Supplementary File 1 [file life-09-00053-s001.pdf]

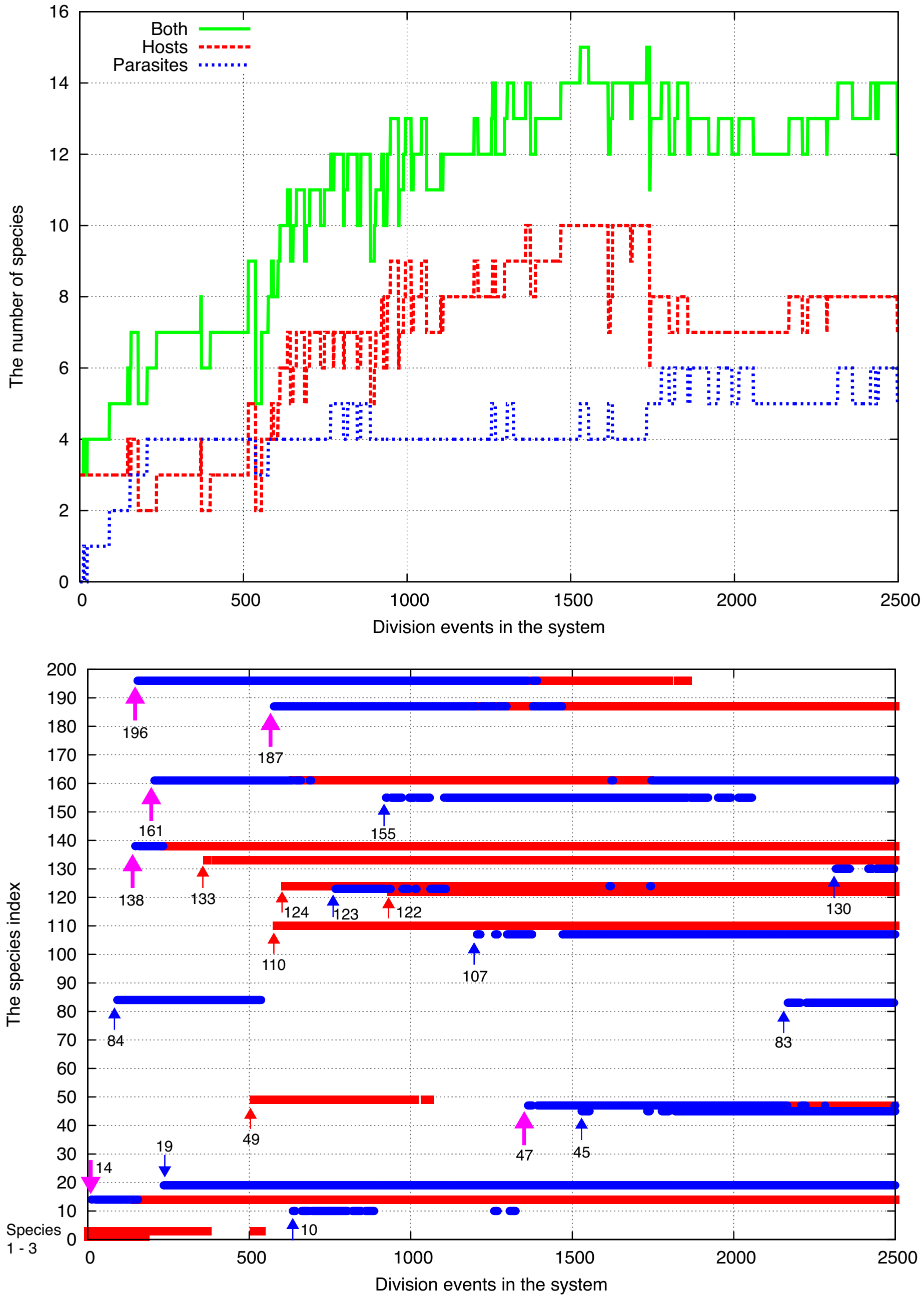

Figure S1: Another example of Figure 5 in the main text. Although we do not distinguish here hosts and sub-hosts, the result indicates that molecular species appear first as parasites, and later they turn to be host species with further diversification of molecular species.
